# Supplementary material for: TGFβ-activated PDHB promotes mitochondrial pyruvate metabolism and contributes to human endoderm differentiation via ATP-dependent BRG1
Source: Nat Commun. 2026 Feb 17;17:2846. doi: 10.1038/s41467-026-69510-0 (PMC13022444; doi:10.1038/s41467-026-69510-0)
Supplement: Supplementary file 1 — Supplementary Information [file 41467_2026_69510_MOESM1_ESM.pdf]

Supplementary Information for

TGFβ-activated PDHB promotes mitochondrial pyruvate metabolism and contributes to human endoderm differentiation via ATP-dependent BRG1

Liming Meng[1,#], Jing Lv[1,2,#], Ying Yi[1,#], Xianchun Lan[1], Chenchao Yan[3], Lihang Zhu[1], Jie Yang[1,4,*], Wei Jiang[1,3,5,*]

[1] Department of Biological Repositories, Frontier Science Center for Immunology and Metabolism, Medical Research Institute, Zhongnan Hospital of Wuhan University, Wuhan University, Wuhan 430071, China;

[2] College of Life Science, Cangzhou Normal University, Cangzhou 061001, China

[3] The Institute of Translational Medicine and Jiangxi Province Key Laboratory of Precision Cell Therapy, The Second Affiliated Hospital, School of Basic Medical Sciences and Institute of Biomedical Innovation, Jiangxi Medical College, Nanchang University, Nanchang 330031, China

[4] State Key Laboratory of Biocatalysis and Enzyme Engineering, School of Life Sciences, Hubei University, Wuhan 430062, China;

[5] Hubei Provincial Key Laboratory of Developmentally Originated Disease, Wuhan 430071, China.

[#] These authors contributed equally.

* To whom correspondence should be addressed. E-mail: jiangw.mri@whu.edu.cn (WJ); jyang@hubu.edu.cn (JY).

Supplementary Figures

Supplementary Figure 1. Glucose metabolism participates in DE differentiation.
Supplementary Figure 2. Effects of BrPA treatment on neuroectodermal and cardiac progenitor differentiation of human ESCs.
Supplementary Figure 3. Metabolism intervention affects the DE differentiation efficiency.
Supplementary Figure 4. SMAD2/3 regulates metabolic genes.
Supplementary Figure 5. The construction and characterization of $PDHB^{+/-}$ cell lines.
Supplementary Figure 6. [U-^{13}C]-glucose metabolic flux analysis reveals enhanced TCA cycle activity following PDHB overexpression.
Supplementary Figure 7. SAM, acetate, α-KG and citrate cannot rescue the differentiation impairment caused by glucose metabolism inhibition.
Supplementary Figure 8. ATP regulates DE differentiation via ATPase activity of BRG1.
Supplementary Figure 9. The expression dynamics of BAF complex and metabolism-related genes during human early development.
Supplementary Figure 10. Reduced ATAC-seq signals at BRG1-bound enhancer regions upon PDHB depletion.
Supplementary Figure 11. The western blot of H3K9/27ac.

Supplementary Tables

Supplementary Table 1: Primers for qRT-PCR analyses
Supplementary Table 2: Primers for ChIP-qPCR analyses

Supplementary Figure 1

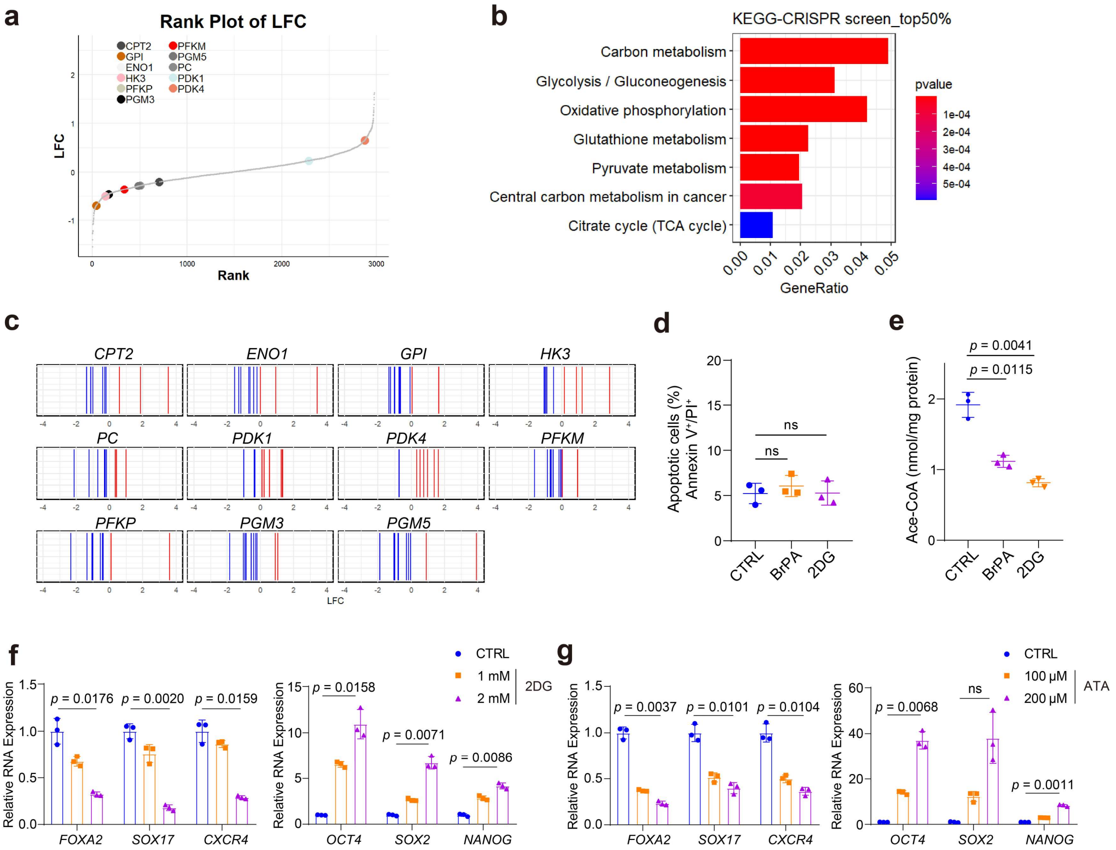

Supplementary Figure 1. Glucose metabolism participates in DE differentiation.

a The log_2(fold-change) (LFC) were calculated for each gene between the top 30% DE cells and the bottom 30% negative cells in the CRISPR screen. The plot shows the distribution of standardized LFC values for the library. The colored dot indicates genes involved in glycolysis and TCA cycle. **b** KEGG results of enriched guides in the top 30% DE cells compared with the bottom 30% negative cells. **c** Different sgRNA LFC scores distribution of genes showed in **a**. **d** Apoptosis was assessed by Annexin V-$FITC^+/PI^+$ staining after BrPA and 2DG treatment (n = 3 independent experiments, F=3.026, P=0.2102, Geisser-Greenhouse corrected). **e** Total acetyl-CoA content in DE cells treated with BrPA or 2DG (n = 3 independent experiments, F=130.4, P=0.0013, Geisser-Greenhouse corrected). **f-g** mRNA levels of ESC markers *OCT4*, *SOX2*, *NANOG,* and DE markers *FOXA2, SOX17, CXCR4* in DE cells treated with 1 mM and 2 mM 2DG (**f**), or with 100 μM and 200 μM ATA (**g**), measured by qPCR (n = 3 independent experiments). Each point represents an individual replicate. Two-way ANOVA for **f** and **g**, followed by Tukey's multiple comparisons test. One-sided hypergeometric test with multiple comparison adjustments was used in **b**. For comparisons between two groups, two-tailed paired t-tests were applied directly. All data are presented as mean ± SD.

Supplementary Figure 2

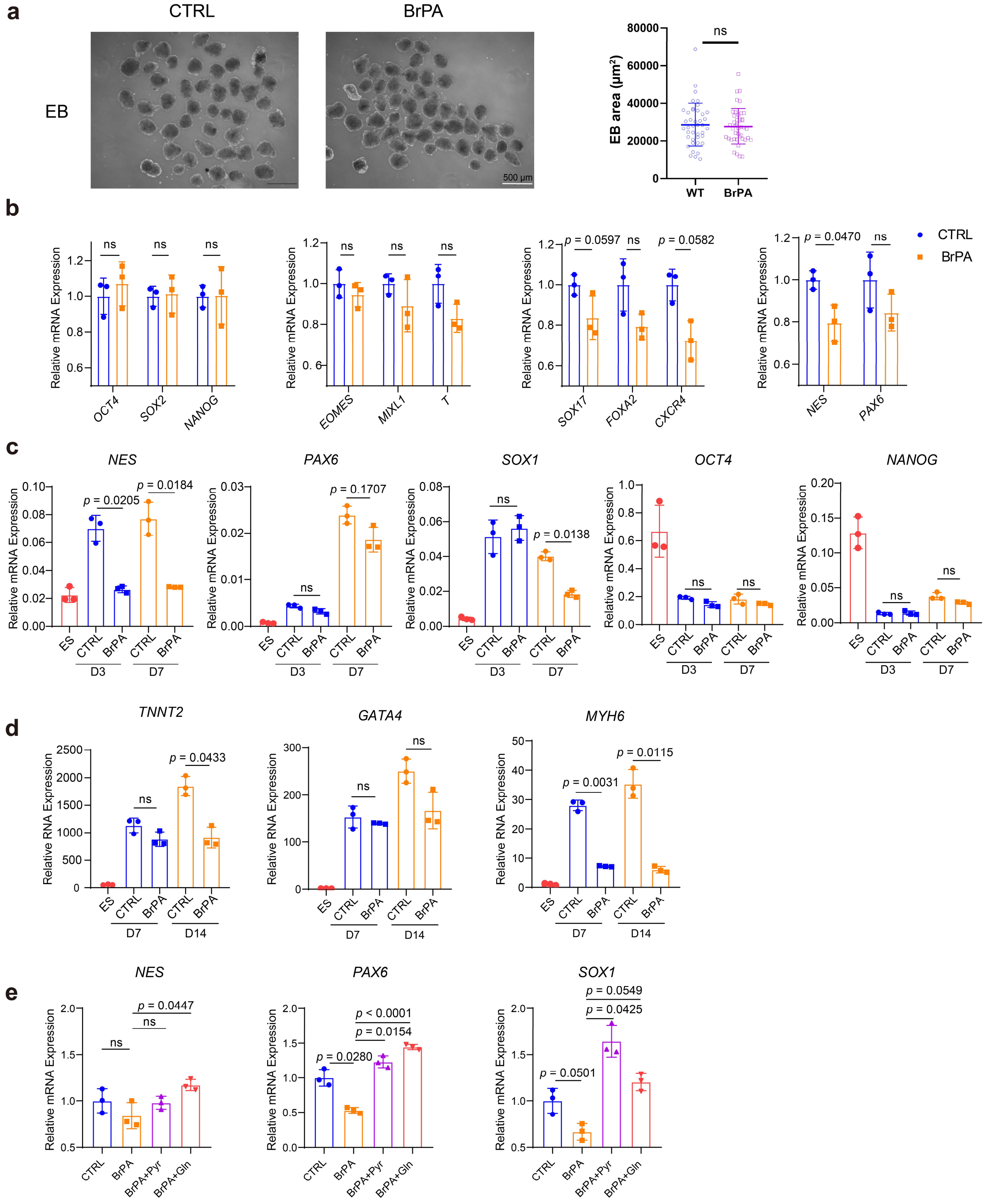

Supplementary Figure 2. Effects of BrPA treatment on neuroectodermal and cardiac progenitor differentiation of human ESCs.

a bright-field images of CTRL and BrPA treated EBs at days 9 in suspension culture, Scale bars, 500 μm (left); scatter graph displayed the area from CTRL and BrPA treated EBs (right). **b** mRNA expression levels of pluripotency markers (*OCT4, SOX2* and *NANOG*) and lineage-specific markers (endoderm: *SOX17, FOXA2, CXCR4*; mesendoderm: *EOMES, MIXL1, T*; ectoderm: *NES, PAX6*) in CTRL and BrPA treated EBs on day 9 (n = 3 independent experiments). **c** Expression changes of pluripotency and neural markers at day 3 and day 7 under BrPA treatment (n = 3 independent experiments). **d** RNA levels of cardiac progenitor markers in BrPA-treated cells (n = 3 independent experiments). **e** mRNA expression levels of ectoderm lineage-specific markers *(NES, PAX6, SOX1*) in CTRL, BrPA and Pyr/Gln rescued EBs on day 9 (n = 3 independent experiments). Each point represents an individual replicate. Statistics were calculated using one-way ANOVA, followed by Dunnett’s multiple comparisons test in **e**. For comparisons between two groups, two-tailed paired t-tests were applied directly. All data are presented as mean ± SD.

Supplementary Figure 3

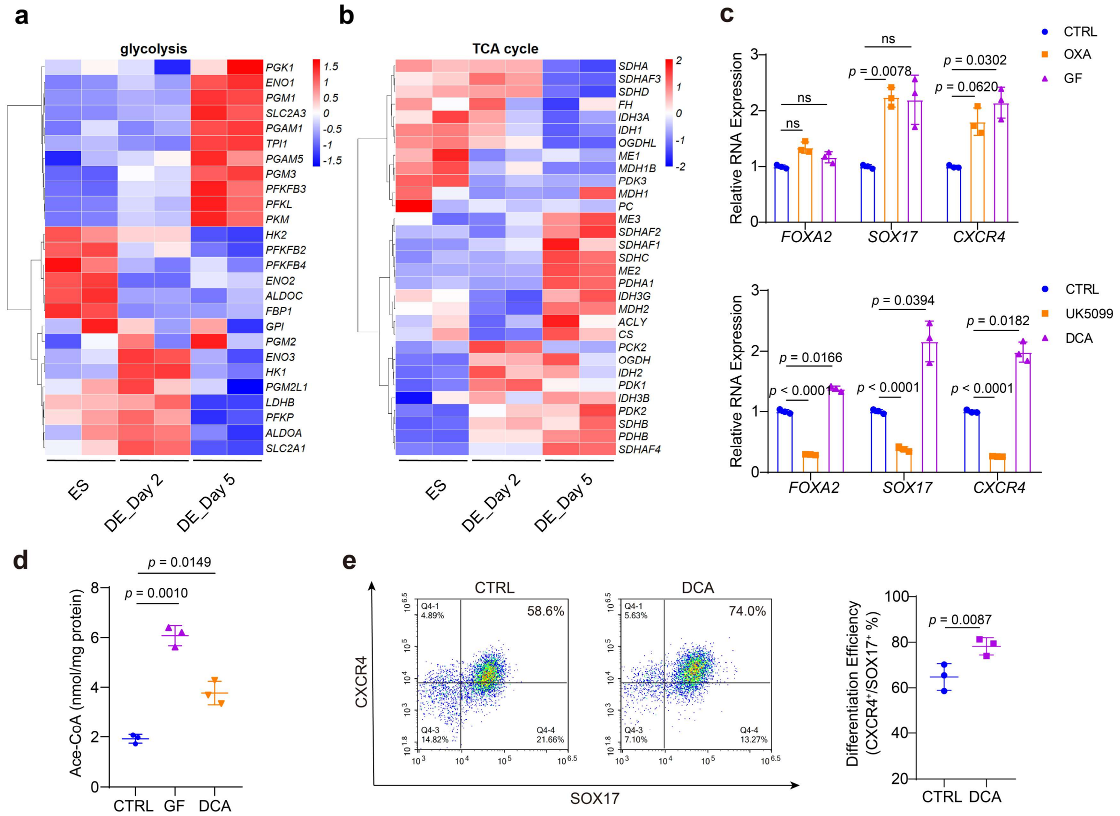

Supplementary Figure 3. Metabolism intervention affects the DE differentiation efficiency.

a-b Heatmap showing gene expression of glycolysis-related genes (**a**) and TCA cycle related genes (**b**) in ESC and DE (Day 2, Day 5) cells. Red denotes higher expression, while blue denotes lower expression. **c** mRNA levels of *FOXA2*, *SOX17* and *CXCR4* in DE cells treated with OXA, GF, UK5099 and DCA in HUES8, measured by qPCR (n = 3 independent experiments). **d** Total acetyl-CoA contents in DE cells treated with GF or DCA (n = 3 independent experiments, F=213.7, P=0.0006, Geisser-Greenhouse corrected). **e** Flow cytometric analysis of SOX17 and CXCR4 showing the DE differentiation efficiency treated with DCA (n = 3 independent experiments). Each point represents an individual replicate. Two-way ANOVA for **c**, followed by Tukey's multiple comparisons test. For comparisons between two groups, two-tailed paired t-tests were applied directly. All data are presented as mean ± SD.

Supplementary Figure 4

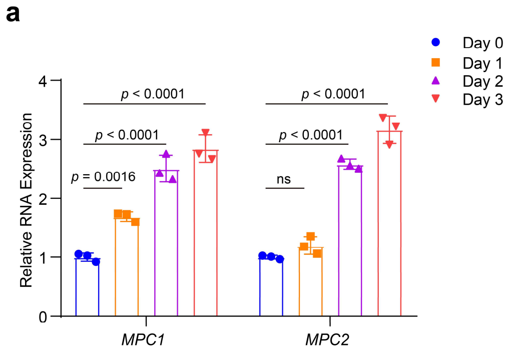

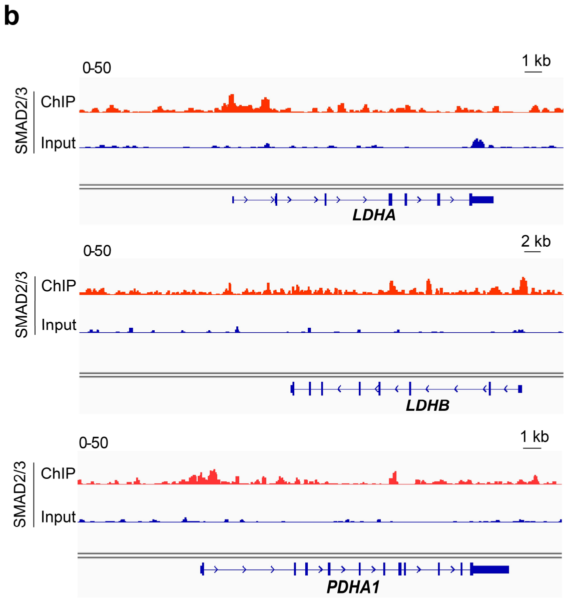

Supplementary Figure 4. SMAD2/3 regulates metabolic genes.

a mRNA levels of *MPC1*, *MPC2* during the DE differentiation process, measured by qRT-PCR (n = 3 independent experiments). **b** IGV plots illustrate the binding levels of SMAD2/3 on *LDHA*, *LDHB* and *PDHA1* locus. Each point represents an individual replicate. Two-way ANOVA for **a**, followed by Tukey's multiple comparisons test. All data are presented as mean ± SD.

Supplementary Figure 5

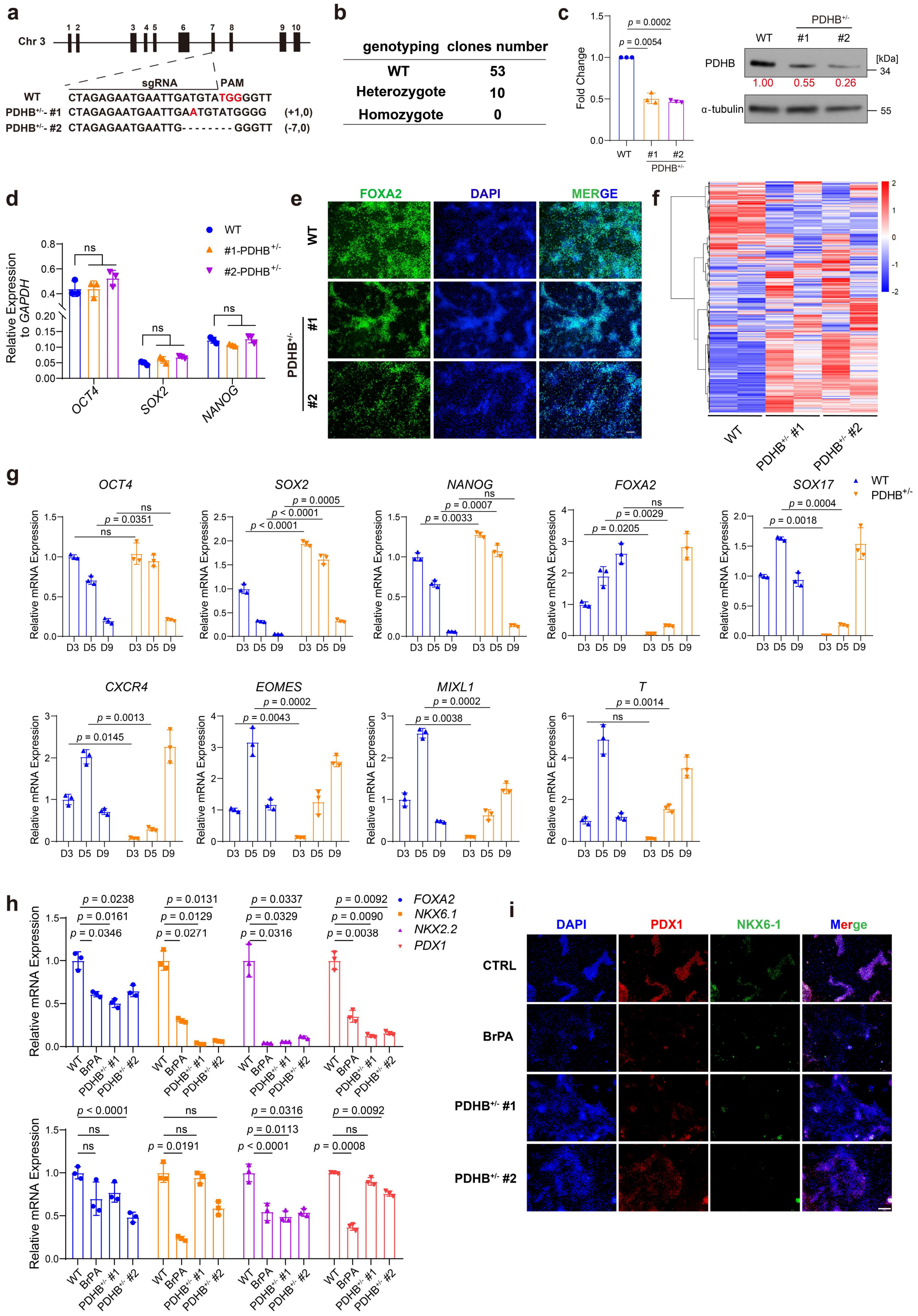

Supplementary Figure 5. The construction and characterization of PDHB$^{+/-}$ cell lines.

a The schematic illustration depicts the PDHB gene locus along with the guide RNA (gRNA) sequence. Below showing the genotypic characteristics of the PDHB$^{+/-}$ clones created using CRISPR-Cas9 technology. **b** Statistics of the clone numbers of PDHB-edited cells. **c** mRNA levels of PDHB in PDHB$^{+/-}$ cell lines, measured by qPCR (left). Protein levels of PDHB between WT and PDHB$^{+/-}$ cell lines (right). (n = 3 independent experiments, F=229.3, P=0.0039, Geisser-Greenhouse corrected). **d** mRNA levels of *OCT4*, *SOX2* and *NANOG* in ES state of WT and PDHB$^{+/-}$ cells, measured by qRT-PCR (n = 3 independent experiments). **e** Immunofluorescence detection of FOXA2 in WT and PDHB$^{+/-}$ DE cells. Scale bars represent 200 μm. **f** Heatmap showing gene expression of differentially expressed genes in WT and PDHB$^{+/-}$ DE cells. Red denotes higher expression, while blue denotes lower expression. **g** mRNA expression levels of pluripotency markers (*OCT4, SOX2* and *NANOG*) and lineage-specific markers (endoderm: *SOX17, FOXA2, CXCR4*; mesendoderm: *EOMES, MIXL1, T*) in CTRL and PDHB$^{+/-}$ EBs on day 3/5/9 (n = 3 independent experiments). **h** mRNA expression levels of pancreatic markers (*FOXA2, NKX6-1, NKX2-2* and *PDX1*) in WT, BrPA treated and PDHB$^{+/-}$ cells on PP1 (top panel) and PP2 (bottom panel) stages (n = 3 independent experiments). **i** Immunofluorescence detection of pancreatic marker PDX1, NKX6-1 in WT, BrPA treated and PDHB$^{+/-}$ cells on PP2 stages. Scale bars represent 200 μm. Each point represents an individual replicate. Two-way ANOVA for **d**, **g** and **h**, followed by Tukey's multiple comparisons test. For comparisons between two groups, two-tailed paired t-tests were applied directly. All data are presented as mean ± SD.

Supplementary Figure 6

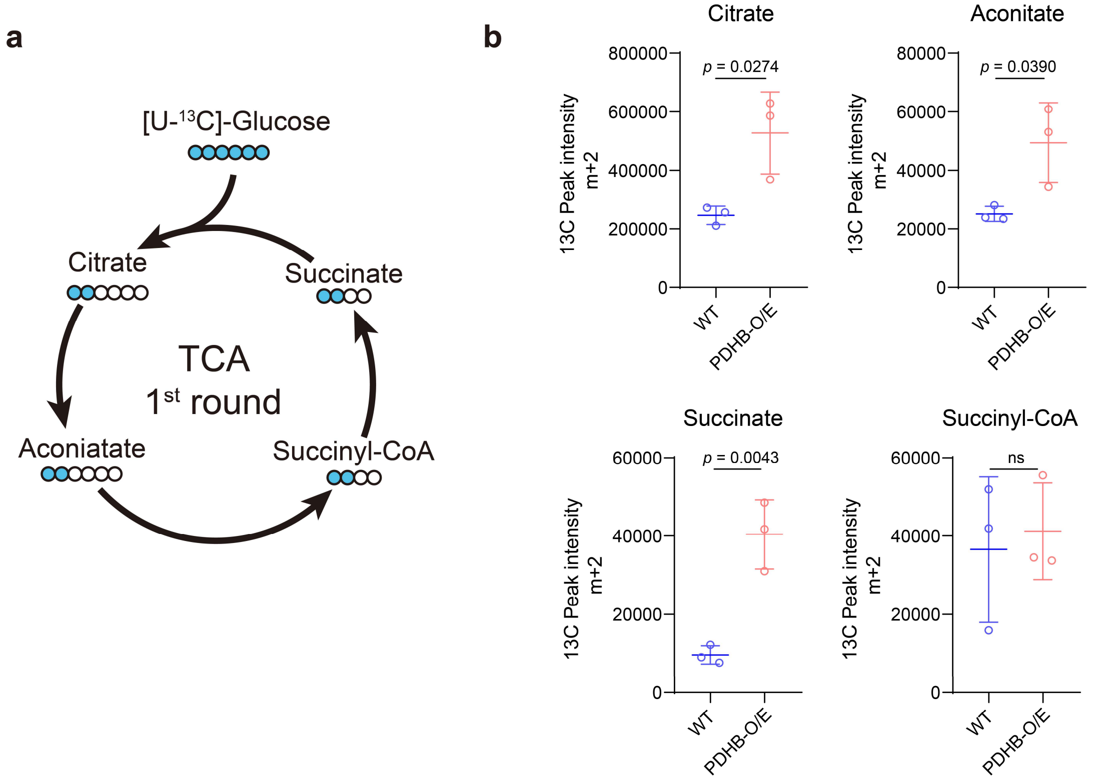

Supplementary Figure 6. [U-^{13}C]-glucose metabolic flux analysis reveals enhanced TCA cycle activity following PDHB overexpression.

a Schematic illustration of the [U-^{13}C]-glucose tracing experiment. **b** Changes in ^{13}C-labeled TCA cycle intermediates after PDHB overexpression, shown as normalized peak intensity (n = 3 independent experiments). Each point represents an individual replicate. For comparisons between two groups, two-tailed unpaired t-tests were applied directly. All data are presented as mean ± SD.

Supplementary Figure 7

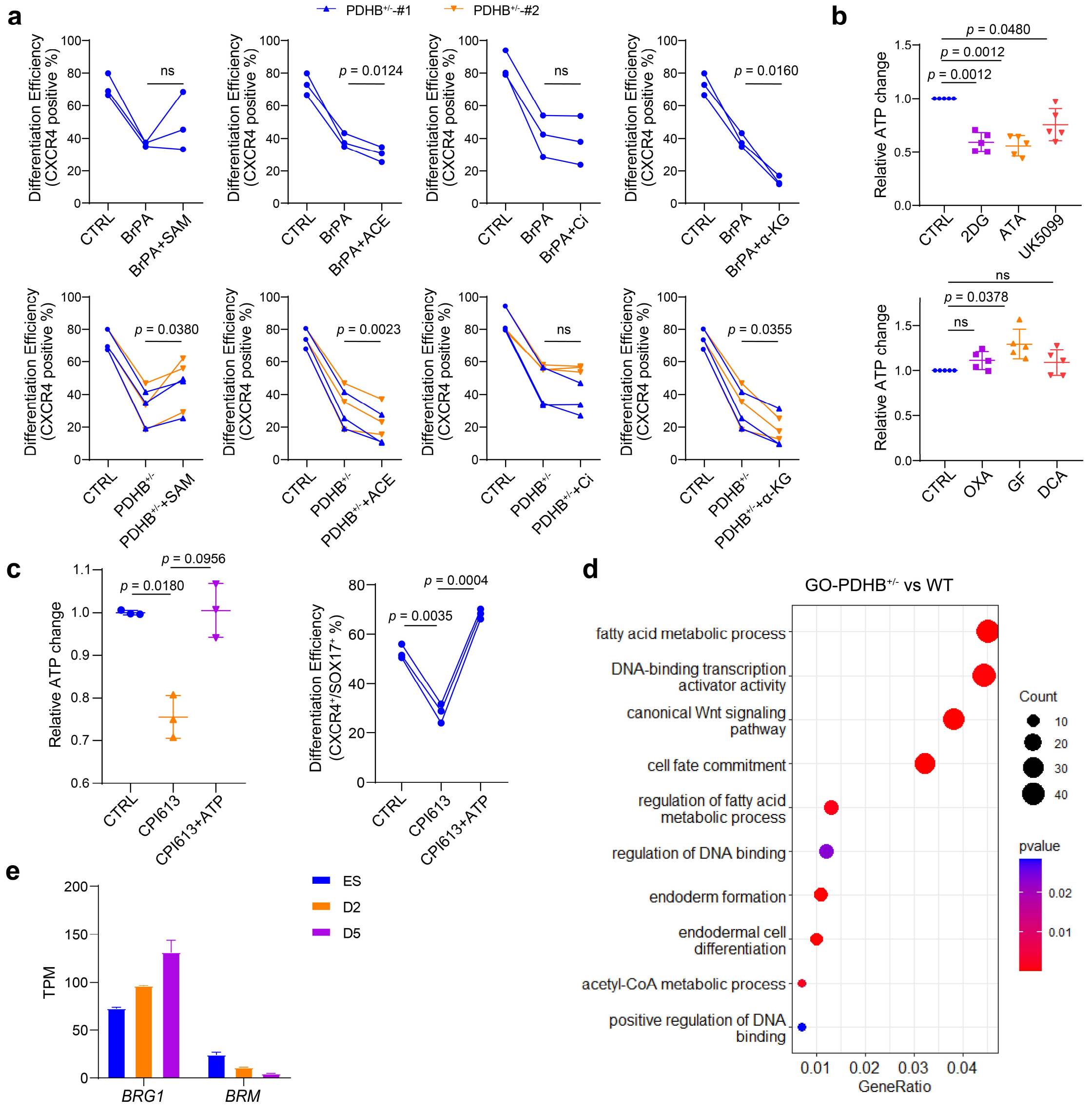

Supplementary Figure 7. SAM, acetate, α-KG and citrate cannot rescue the differentiation impairment caused by glucose metabolism inhibition.

a Flow cytometric analysis of CXCR4 showing the DE differentiation efficiency upon BrPA or in $PDHB^{+/-}$ cells with or without exogenous SAM, acetate, α-KG and citrate. (n = 3 independent experiments). **b** Total ATP content in DE cells treated with 2DG, ATA, UK, DCA, OXA and GF (n = 5 independent experiments). **c** Measurement of ATP content and flow cytometric analysis of SOX17 and CXCR4 expression in control and CPI613 treated groups, with or without ATP supplementation (n = 3 independent experiments). **d** GO enrichment analysis of differentially expressed genes in WT and $PDHB^{+/-}$ DE cells. **e** RNA levels of *BRG1* and *BRM* in RNA-seq data from ES, day 2/5 during DE differentiation (presented with TPM values). Each point represents an individual replicate. Statistics were calculated using one-way ANOVA, followed by Dunnett's multiple comparisons test in **b** and **c**. One-sided hypergeometric test with multiple comparison adjustments was used in **d**. For comparisons between two groups, two-tailed paired t-tests were applied directly. All data are presented as mean ± SD.

Supplementary Figure 8

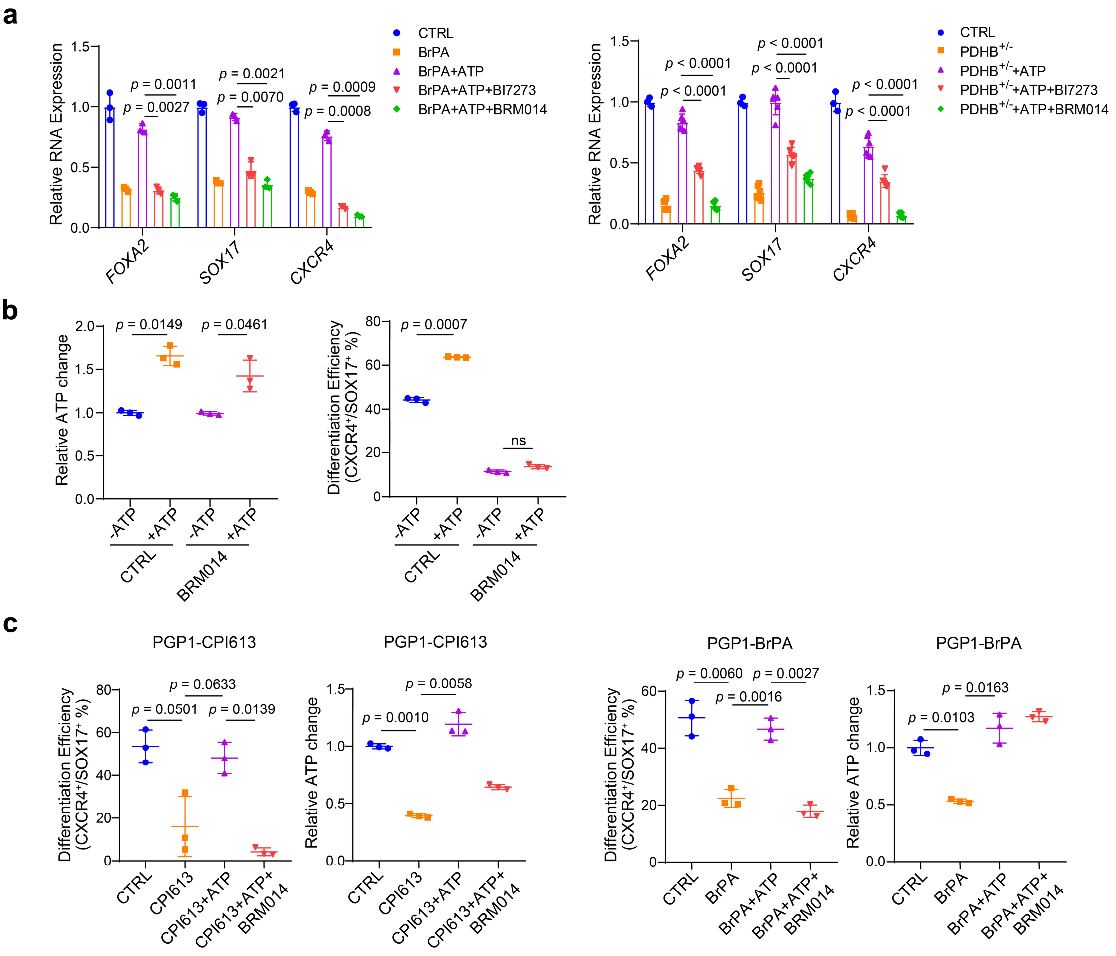

Supplementary Figure 8. ATP regulates DE differentiation via ATPase activity of BRG1.

a mRNA levels of *FOXA2*, *SOX17* and *CXCR4* in DE cells treated with BRM014 or BI7273, together with exogenous ATP, measured by qPCR (n = 3 independent experiments). **b** Measurement of ATP content and flow cytometric analysis of SOX17 and CXCR4 expression in control and BRM014 treated cells, with or without ATP supplementation (n = 3 independent experiments). **c** Measurement of intracellular ATP content and flow cytometric analysis of SOX17 and CXCR4 expression in PGP1 cells, the groups include CPI613/BrPA treatment, ATP rescue and BRM014 block (n = 3 independent experiments). Each point represents an individual replicate. For comparisons between two groups, two-tailed paired t-tests were applied directly. All data are presented as mean ± SD.

Supplementary Figure 9

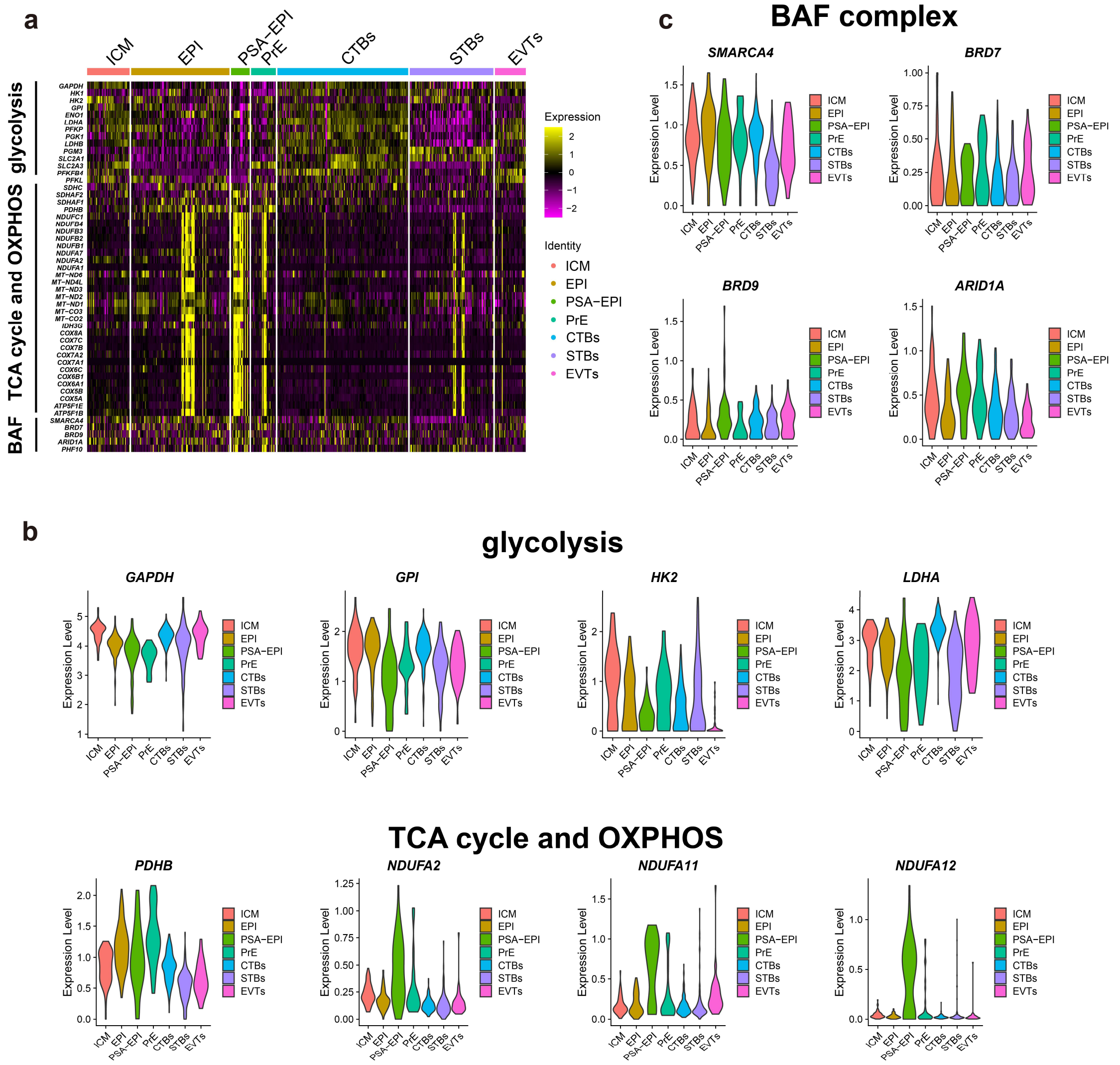

Supplementary Figure 9. The expression dynamics of BAF complex and metabolism-related genes during human early development.

a Different clusters expressed metabolism and BAF associated genes (CTBs: cytotrophoblasts; STBs: syncytiotrophoblasts; EVTs: extravillous cytotrophoblasts; PrE: primitive endoderm/hypoblast). **b-c** Violin plot showing expression of metabolism and BAF associated genes.

Supplementary Figure 10

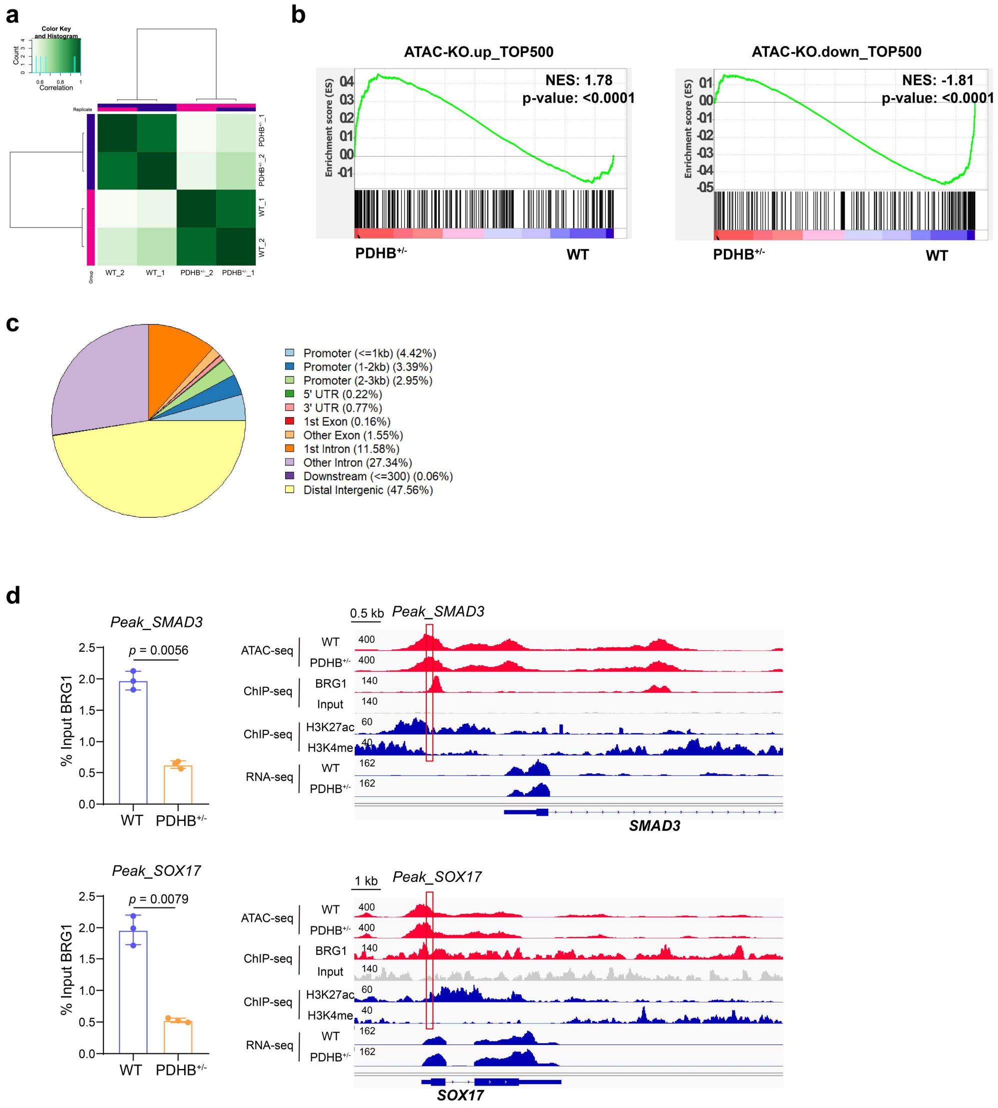

Supplementary Figure 10. Reduced ATAC-seq signals at BRG1-bound enhancer regions upon PDHB depletion.

a Clustering analysis results of WT and $PDHB^{+/-}$ ATAC-seq. **b** GSEA profile of top 500 differentially accessible genes from ATAC-seq. **c** Distribution of BRG1-binding sites across genomic regions. UTR, untranslated region. **d** ChIP-qPCR of BRG1 at *SMAD3* and *SOX17* loci in wild-type and $PDHB^{+/-}$ cells (left). IGV tracks showing BRG1, H3K27ac, H3K4me binding, and ATAC-seq and RNA-seq signals in wild-type and $PDHB^{+/-}$ cells at *SMAD3* and *SOX17* loc (right). Each point represents an individual replicate. The permutation-based, one-sided test was performed in **b**. For comparisons between two groups, two-tailed paired t-tests were applied directly. All data are presented as mean ± SD.

Supplementary Figure 11

a

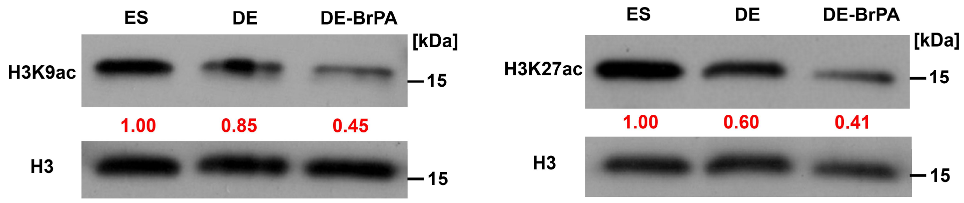

Supplementary Figure 11. The western blot of H3K9/27ac.

a Protein levels of H3K9/27ac upon BrPA treated in DE cells. The values shown in the figure represent the results after normalization of the target protein to the loading control, followed by further standardization.

Supplementary Table 1: Primers for qRT-PCR analyses

Primer name	Forward primer	Reverse primer
GAPDH	AATGAAGGGGTCATTGATGG	AAGGTGAAGGTCGGAGTCAA
OCT4	CAAAGCAGAAACCCTCGTGC	TCTCACTCGGTTCTCGATACTG
SOX2	GTCATTTGCTGTGGGTGATG	AGAAAAACGAGGGAAATGGG
NANOG	CCCCAGCCTTTACTCTTCCTA	CCAGGTTGAATTGTTCCAGGTC
FOXA2	GGAGCAGCTACTATGCAGAGC	CGTGTTCATGCCGTTCATCC
SOX17	GCATGACTCCGGTGTGAATCT	TCACACGTCAGGATAGTTGCAGT
CXCR4	TACACCGAGGAAATGGGCTCA	AGATGATGGAGTAGATGGTGGG
PDHA1	GCTTCTCAAGGACAGGATGG	CTCTTCCAAAGGTGGCTCAG
PDHB	AAGAGGCGCTTTCACTGGAC	ACTAACCTTGTATGCCCCATCA
LDHA	ATGGCAACTCTAAAGGATCAGC	CCAACCCCAACAACTGTAATCT
LDHB	TGGTATGGCGTGTGCTATCAG	TTGGCGGTCACAGAATAATCTTT
BRG1	GACCAGCACTCCCAAGGTTAC	CTGGCCCGGAAGACATCTG
BRM	AGGGGATTGTAGAAGACATCCA	TTGGCTGTGTTGATCCATTGG
EOMES	CACATTGTAGTGGGCAGTGG	CGCCACCAAACTGAGATGAT
MIXL1	GAGACTTGGCACGCCTGT	GGTACCCCGACATCCACTT
T	GATGATCGTGACCAAGAACGG	CCACGAAGTCCAGCAGGAA
NES	GAGGGAAGTCTTGGAGCCAC	AAGATGTCCCTCAGCCTGG
PAX6	TCCGTTGGAACTGATGGAGT	GTTGGTATCCGGGGACTTC
SOX1	ATTATTTTGCCCGTTTTCCC	TCAAGGAAACACAATCGCTG
TNNT2	AAGAGGCAGACTGAGCGGGAAA	AGATGCTCTGCCACAGCTCCTT
GATA4	CAGGCGTTGCACAGATAGTG	CCCGACACCCCAATCTC
MYH6	GGAAGACAAGGTCAACAGCCTG	TCCAGTTTCCGCTTTGCTCGCT
MPC1	ACTATGTCCGAAGCAAGGATTTC	CGCCCACTGATAATCTCTGGAG
MPC2	TACCACCGGCTCCTCGATAAA	TATCAGCCAATCCAGCACACA
NKX6.1	AGACCCACTTTTTCCGGACA	CCAACGAATAGGCCAAACGA
NKX2.2	GTCAGGGACGGCAAACCAT	GCGCTGTAGGCAGAAAAGG
PDX1	TTAGGATGTGGACGTAATTCCTGTT	GGCCACTGTGCTTGTCTTCA

Supplementary Table 2: Primers for ChIP-qPCR analyses

Primer name	Forward primer	Reverse primer
Peak_TCF4	CTCCCTGTGGCTCAGTCTTG	AGTGGATGGAGGTTGCTTGG
Peak_FOXI1	ACAGGGGGTCTCTGTGTGAT	AAGGAAGTCCCACGCTTCTG
Peak_Neg	CCATAATTTCTGTCCCCAGACTC	GAAGATGGCAATAGCAGGAGAG
Peak_SMAD3	CCCACTTGAGGGTTCCAGATA	CCGACTAGCCGGTGTCTAA
Peak_SOX17	TGGGTACGCTGTAGACCAGA	GCACTGAGATGCCCCGAG
PDHB peak1	ACTGGGTGCTCAATTGTCCC	TGGCCATAGGGACAACCTCT
PDHB peak2	TTGGCTTCCACAACCACTCTTAT	CAGGTTATACATGGATGCAAAG
PDHB negative	TACCATGACCAGCAGCCTTG	GCCAGCTAGGTAAAGGGGAC
