# Supplementary material for: TGFβ-activated PDHB promotes mitochondrial pyruvate metabolism and contributes to human endoderm differentiation via ATP-dependent BRG1
Source: Nat Commun. 2026 Feb 17;17:2846. doi: 10.1038/s41467-026-69510-0 (PMC13022444; doi:10.1038/s41467-026-69510-0)
Supplement: Supplementary file 2 — Description of Additional Supplementary Files [file 41467_2026_69510_MOESM2_ESM.pdf]

Description of Additional Supplementary Files

File Name: Supplementary Data 1

Description: Raw data of [U-^{13}C]-glucose traced central carbon metabolism flux experiments (glycolysis, tricarboxylic acid cycle, pentose phosphate pathway) in control and PDHB-OE groups.
